# The Potential Anti-Cancer Effects of Polish Ethanolic Extract of Propolis and Quercetin on Glioma Cells Under Hypoxic Conditions

**DOI:** 10.3390/molecules30143008

**Published:** 2025-07-17

**Authors:** Małgorzata Kłósek, Anna Kurek-Górecka, Radosław Balwierz, Grażyna Pietsz, Zenon P. Czuba

**Affiliations:** 1 Department of Microbiology and Immunology, Faculty of Medical Sciences, Medical University of Silesia in Katowice, Jordana 19, 41-808 Zabrze, Poland; gpietsz@sum.edu.pl (G.P.); zczuba@sum.edu.pl (Z.P.C.); 2 Institute of Chemistry, University of Opole, Oleska 48, 45-052 Opole, Poland; radoslaw.balwierz@uni.opole.pl

**Keywords:** propolis, quercetin, cytokine, hypoxia, astrocytes, glioma cells

## Abstract

Tissue hypoxia is commonly observed in head cancers and contributes to both molecular and functional changes in tumour cells. It is known to stimulate erythropoiesis, angiogenesis, and metabolic alterations within tumour cells. Glioblastoma, a type of brain tumour, is characterized by rapid proliferation and aggressive growth. Recent studies have indicated that natural products may hold potential as components of cancer therapy. Among these, Polish propolis and its active compound, quercetin, have demonstrated promising anti-cancer properties. The aim of this study was to evaluate the concentrations of selected cytokines—specifically IL-6, IL-9, vascular endothelial growth factor (VEGF), platelet-derived growth factor (PDGF-BB), interferon gamma-induced protein 10 (IP-10), and monocyte chemoattractant protein-1 (MCP-1)—produced by astrocytes of the CCF-STTG1 cell line. The cytotoxic effects of ethanolic extract of propolis (EEP) and quercetin were assessed using the MTT assay. Astrocytes were stimulated with lipopolysaccharide (LPS, 200 ng/mL) and/or IFN-α (100 U/mL), followed by treatment with EEP or quercetin (25–50 µg/mL) under hypoxic conditions for two hours. Cytokine concentrations were measured using the xMAP Luminex Multiplex Immunoassay and the Multiplex Bead-Based Cytokine Kit. Our study demonstrated that Polish propolis and its component quercetin modulate the tumour microenvironment in vitro, primarily by altering the levels of specific cytokines. The HCA analysis revealed that IL-6 and MCP-1 formed a distinct cluster at the highest linkage distance (approximately 100% of Dmax), suggesting that their expression patterns are significantly different from those of the other cytokines and that they are more similar to each other than to the rest. PCA analysis showed that EEP-PL (50 μg/mL) with IFN-α and EEP-PL (50 μg/mL) with LPS exert similar activities on cytokine secretion by astrocytes. Similar effects were demonstrated for EEP-PL 50 μg/mL + LPS + IFN-α, EEP-PL 25 μg/mL + IFN-α and EEP-PL 25 μg/mL + LPS + IFN-α. Our findings suggest that Polish propolis and quercetin may serve as promising natural agents to support the treatment of stage IV malignant astrocytoma. Nonetheless, further research is needed to confirm these results.

## 1. Introduction

Gliomas are the most common type of brain tumour, accounting for approximately 60% of all tumours in this region. They are highly malignant, with an average survival of 14–15 months for treated patients and only 2–4 months for those untreated. As a result, gliomas have the lowest survival rate among central nervous system (CNS) tumours [1]. Histopathologically, gliomas are classified based on the type of glial cells from which they originate. These include astrocytomas, ependymomas, oligodendrogliomas, and mixed glial neoplasms. Astrocytomas arise from star-shaped astrocytes, and ependymomas develop from the cells lining the ventricles (lining glia), while oligodendrogliomas and mixed glial tumours originate from multiple glial cell types, primarily astrocytes or oligodendrocytes. According to the World Health Organization (WHO), the classification of CNS tumours has evolved from a purely histological approach to a multilayered system that incorporates molecular biomarkers. This integrated method is essential for accurate diagnosis, risk stratification, and the identification of prognostic and predictive clinical targets [2]. Currently, glial, glioneuronal, and neuronal tumours are grouped into a distinct category and classified into six subtypes: adult-type diffuse gliomas, paediatric-type diffuse gliomas of low malignancy, paediatric-type diffuse gliomas of high malignancy, circumscribed astrocytic gliomas, glioneuronal tumours, and a sixth group known as ependymal tumours. Additionally, gliomas are further classified based on their degree of malignancy [3].

Grade I gliomas are the least aggressive, while Grade IV represents the most malignant form. A major factor contributing to the poor survival rate in glioblastoma patients is the presence of hypoxic tissue surrounding the tumour. This hypoxia is linked to neovascularization, necrosis, cell migration, and metabolic reprogramming. A group of transcription factors known as hypoxia-inducible factors (HIFs) plays a central role in regulating the expression of genes responsive to low oxygen levels. Tumour hypoxia is believed to increase the risk of metastasis, tumour recurrence, resistance to chemotherapy and radiotherapy, and enhanced invasiveness—all of which contribute to decreased patient survival [4]. Considering this aspect, the experiment was conducted under hypoxic conditions.

Natural products play a significant role in the development of cancer treatments. Vincristine and vinblastine are anti-cancer compounds and among the most important plant-derived alkaloids, produced by *Catharanthus roseus* (L.). Vincristine is a key component of the PCV regimen (procarbazine, lomustine, vincristine), which is commonly used to treat primary brain tumours [5]. In contrast, vinblastine monotherapy is considered the standard first-line chemotherapy for paediatric low-grade glioma (pLGG) patients by the Canadian group and is now also widely used as an initial treatment in many centres across Europe [6].

Natural products like propolis and quercetin, which belongs to flavonoids occuring in propolis exhibit an anticancer activity. Scientists emphasise that propolis extracts can inhibit cancer growth by affecting cell proliferation, angiogenesis and invasion. Quercetin induces both apoptosis and necrosis by decreasing the expression of thymidylate synthase (TS), an enzyme active during the S-phase of the cell cycle, and also triggers caspase-3-dependent apoptosis [7,8,9].

Propolis, also known as bee glue, is a natural, viscous, resinous substance produced by bees from plant resins collected from the buds and young shoots of trees and other plants, combined with beeswax and their own glandular secretions. Polish propolis is derived primarily from the leaf buds of black poplar (*Populus nigra*). Numerous studies have demonstrated that propolis possesses a range of valuable medicinal properties, including antibacterial, anti-inflammatory, and anticancer effects [10,11,12,13,14]. The aim of our study was to evaluate the effects of propolis and its active component, quercetin, on the concentrations of selected cytokines released by astrocytes. We aimed to investigate how propolis and its compound—quercetin—might influence the tumour microenvironment, particularly through their impact on the concentration of selected cytokines, given the well-established role of inflammation in carcinogenesis. To this end, we measured the levels of cytokines crucial to cancer growth and progression in central nervous system tumours such as astrocytoma. Specifically, we examined the concentrations of IL-6, VEGF, PDGF-BB, IP-10, MCP-1, and IL-9 secreted by the CCF-STTG1 cell line.

Cytokines are a family of protein molecules that play a key role in carcinogenesis. Within the tumour microenvironment, cytokines contribute to processes such as invasion, metastasis, and immune suppression. The ‘cytokine field’ concept describes how elevated cytokine levels in the tumour microenvironment create a field effect, extending their influence to surrounding cells. The release of cytokines into body fluids also affects immune responses and inflammation associated with cancer. The novelty of this study lies in examining the impact of Polish ethanolic extract of propolis and its active component, quercetin, on the concentrations of specific cytokines linked to tumour proliferation, progression, and metastasis. However, it should be emphasized that, in general, grade IV astrocytoma does not exhibit metastatic potential, but is characterized by rapid infiltrative growth. This type of growth makes complete surgical resection challenging, which underscores the importance of adjuvant therapies such as chemotherapy and radiotherapy. Furthermore, the hypoxic conditions commonly found in the tumour microenvironment—also observed in head and neck cancers—contribute to treatment resistance. Taking this into consideration, we decided to conduct our study under hypoxic conditions.

## 2. Results

### 2.1. Viability of Astrocyte Cell Line CCF-STTG1 After Stimulation with an Ethanolic Extract of Polish Propolis (EEP PL) and Quercetin in Hypoxia Conditions

**Figure 1:**
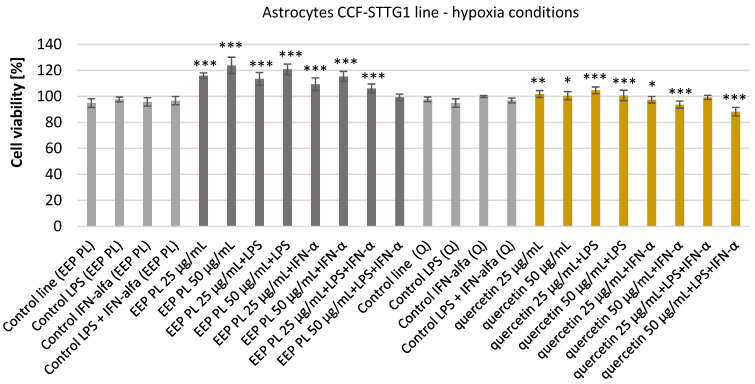
Viability [%] of astrocyte cell line CCF-STTG1 under hypoxic conditions after treatment with ethanolic extract of Polish propolis (EEP-PL) or quercetin (Q), alone or in combination with lipopolysaccharide (LPS, 200 ng/mL), interferon-alpha (IFN-α, 100 U/mL), or both (LPS + IFN-α). Cells were incubated with EEP-PL or quercetin at final concentrations of 25 and 50 µg/mL. Cell viability was assessed by MTT assay, and results are expressed as mean ± standard deviation (SD) from three independent experiments (n = 3). Statistical significance was evaluated using the two-tailed Student’s *t*-test, with comparisons made between treated groups and their respective stimulated controls (i.e., EEP + LPS vs. LPS alone, Q + IFN-α vs. IFN-α alone, etc.). Statistical analysis was performed using STATISTICA v13.1 software (StatSoft, Tulsa, OK, USA); * *p* < 0.05, ** *p* < 0.01, *** *p* < 0.001 vs. *corresponding control group*.

The astrocyte cells were incubated with an ethanolic extract of Polish propolis (EEP PL) and quercetin at the final concentrations of 25–50 μg/mL for 24 h. EEP PL alone at both tested concentrations caused increases the viability of tested cells from 94.86 ± 3.35% to 123.88 ± 6.25% incubated under hypoxia conditions (Figure 1). EEP PL with LPS and EEP PL with IFN-α resulted in a significant increase in the viability of the astrocytes at both concentrations to 120.83 ± 4.03% and to 115.41 ± 3.80% accordingly. EEP PL at the final concentration of 25 μg/mL in combination with LPS + IFN-α caused significant increase the viability from 96.73 ± 3.21% to 106.11 ± 3.43%.

After incubating astrocyte cells with quercetin, the viability was increased from 97.71 ± 1.81% to 101.85 ± 2.63% and to 100.50 ± 3.20% accordingly. Quercetin with LPS also increased the viability of tested cells in both concentrations used. Quercetin with IFN-α and with LPS + IFN-α caused decreases in the viability of tested cells to 93.72 ± 2.68% and to 88.26 ± 3.18% accordingly. Neither quercetin nor propolis had an effect of reducing the viability of the tested cells.

### 2.2. Effect of Polish Ethanolic Extracts of Propolis and Quercetin on Selected Cytokine Production by Cell CCF-STTG1 Line of Astrocytes by LPS, IFN-α, and LPS + IFN-α in Hypoxia Conditions

**Figure 2:**
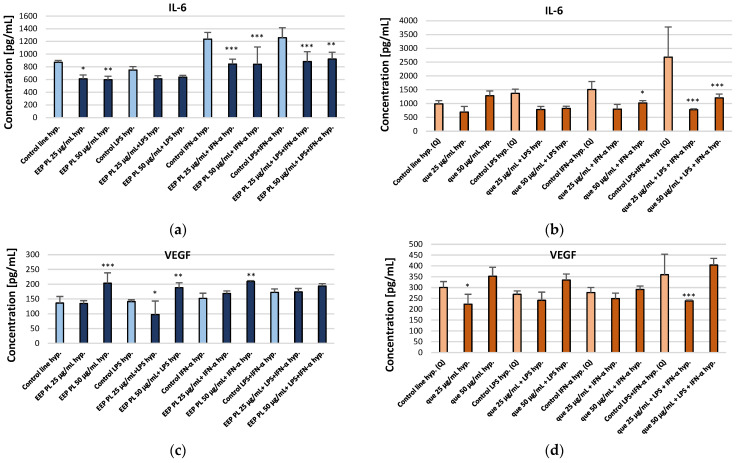

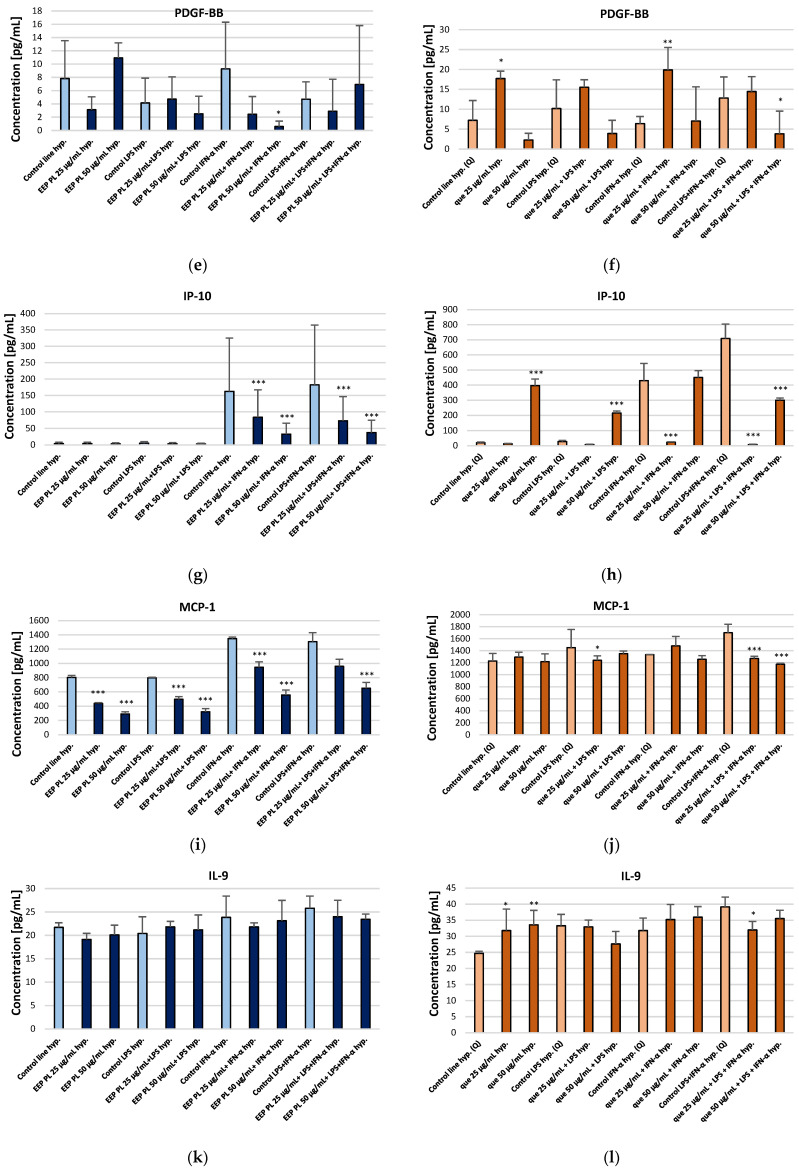
Concentrations of IL-6 (**a**,**b**), VEGF (**c**,**d**), PDGF-BB (**e**,**f**), IP-10 (**g**,**h**), MCP-1 (**i**,**j**), and IL-9 (**k**,**l**) [pg/mL] secreted by CCF-STTG1 astrocytes cultured under hypoxic conditions and treated with Polish ethanolic extract of propolis (EEP-PL) or quercetin (Q), at 25 and 50 µg/mL in combination with IFN-α, LPS, or both. Data are expressed as mean ± SD of three independent experiments. Statistical significance was determined using one-way ANOVA followed by Fisher’s LSD post hoc test (* *p* < 0.05, ** *p* < 0.01, *** *p* < 0.001) in STATISTICA 13.1 software. Comparisons were made versus the respective stimulated control groups (LPS, IFN-α or LPS + IFN-α alone). Further details can be found in the Supplementary Materials.

We examined the activity of EEP PL and quercetin on the production of IL-6, VEGF, PDGF-BB, IP-10, MCP-1 and IL-9 by the CCF-STTG1 cell line present in the tumour microenvironment of CNS. The effects of EEP PL and quercetin were examined in experimental settings using LPS, IFN-α, and a combination of both. The experimental findings are shown in Figure 2 and detailed in the Supplementary Materials. Drawing on prior research and our earlier study [12,15], it was anticipated that LPS, IFN-α, and their combination would enhance the secretion of the cytokines relative to the control cell line. This was also observed in this experiment. Considering the effect of EEP PL on the concentration of selected cytokines, it was observed that EEP-PL, both at the concentrations tested and in combinations with IFN-α and LPS + IFN-α, caused a reduction in IL-6 levels under hypoxic conditions (Figure 2a). For EEP, PL levels of IL-6 decreased from 872.09 ± 30.41 pg/mL to 611.32 ± 61.78 pg/mL and to 597.75 ± 53.42 pg/mL, respectively. Quercetin at the final concentrations of 25 μg/mL in combination with IFN-α and quercetin with LPS + IFN-α, resulted in a decrease IL-6 levels under hypoxic conditions to 798.57 ± 171.48 pg/mL and to 779.40 ± 29.65 pg/mL, respectively. Moreover, quercetin at concentration of 50 μg/mL in combination with LPS and IFN-α caused a decrease in IL-6 levels from 2683.48 ± 1093.40 pg/mL to 1206.23 ± 135.63 pg/mL (Figure 2b). EEP-PL at a final concentration of 25 μg/mL with LPS decreased the concentrations of VEGF from 136.51 ± 21.95 pg/mL to 97.57 ± 45.61 pg/mL. However, 50 μg/mL of EEP-PL, 50 μg/mL of EEP-PL + LPS, and 50 μg/mL of EEP-PL + IFN-α caused an increased the concentrations of VEGF released by astrocytes to 203.38 ± 35.04 pg/mL, 188.51 ± 16.39 pg/mL and 209.46 ± 2.91 pg/mL, respectively (Figure 2c). Quercetin at the final concentrations of 25 μg/mL alone and in combination with LPS + IFN-α, resulted in a decrease of VEGF levels to 223.59 ± 45.51 pg/mL and to 238.74 ± 3.25 pg/mL, respectively (Figure 2d). A statistically significant decrease in PDGF-BB levels was observed only with 50 μg/mL EEP-PL combined with IFN-α, from 11.63 ± 9.29 pg/mL to 0.58 ± 0.82 pg/mL (Figure 2e). Quercetin at the final concentration of 25 μg/mL alone and in combination with IFN-α, resulted in an increase PDGF-BB to 17.71 ± 1.88 pg/mL and to 19.88 ± 5.65 pg/mL, respectively. In contrast, 50 μg/mL of quercetin with LPS + IFN-α caused a decrease in the concentration of PDGF-BB to 3.83 ± 5.70 pg/mL (Figure 2f). EEP-PL at both tested concentrations combined with IFN-α and with LPS + IFN-α caused IP-10 to decrease to 33.01 ± 10.80 pg/mL and to 37.81 ± 3.89 pg/mL (Figure 2g). Quercetin at the final concentrations of 50 μg/mL in combination with of LPS and/or IFN-α decreased IP-10 (Figure 2h). EEP PL, at both tested concentrations and in all combinations, resulted in a decrease in MCP-1 secreted by astrocytes (Figure 2i). The decreased MCP-1 concentration was affected by quercetin at a concentration of 25 μg/mL with LPS and with LPS + IFN-α and also by quercetin at a concentration of 50 μg/mL with both stimulators (Figure 2j). EEP-PL, at each of the concentrations tested and in all variants, did not statistically significantly affect IL-9 secretion by astrocytes of the CCF-STTG1 lineage (Figure 2k). An IL-9 decrease was observed for quercetin at a concentration of 25 μg/mL alone and with LPS and for quercetin at a concentration of 50 μg/mL with IFN-α (Figure 2l).

### 2.3. Comparative Effects of EEP-PL on IL-6, VEGF, PDGF-BB, MCP-1, IP-10 and IL-9 Secretion in LPS and/or IFN-α Induced by Astrocyte CCF-STTG1 Cell Line

**Figure 3:**
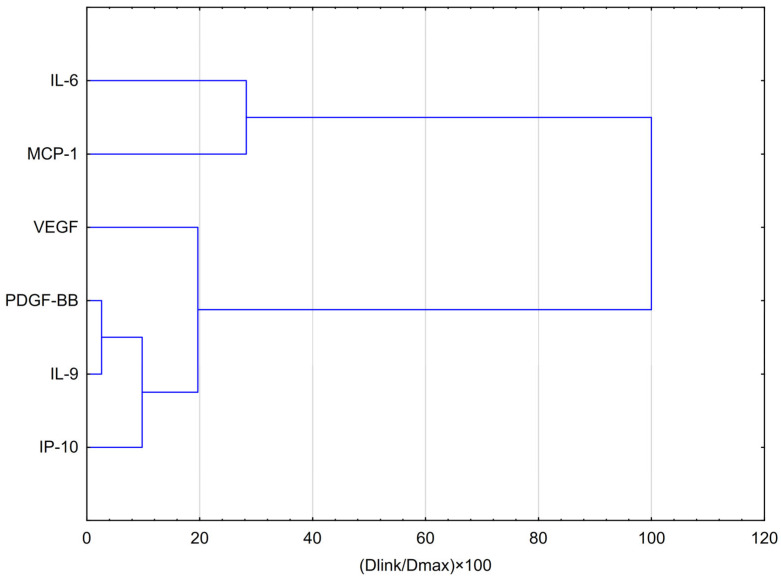
Dendrogram obtained via HCA analysis of data regarding the behaviour of IL-6, VEGF, PDGF-BB, MCP-1, IP-10 and IL-9 secretion by CCF-STTG1 cells stimulated by LPS, IFN-α and LPS + IFN-α treatment with EEP-PL in hypoxia conditions. Dlink denotes the linkage distance, or the measured separation between two clusters. Dmax indicates the maximum possible distance that may occur between any clusters.

HCA analysis (Figure 3) showed that VEGF formed part of this group at a slightly greater distance, suggesting a partially similar response pattern. In contrast, IL-6 and MCP-1 clustered separately at the highest linkage distance (~100% of Dmax), indicating that their expression profiles differ substantially from those of the other cytokines and are more closely related to each other than to the remaining variables.

**Figure 4:**
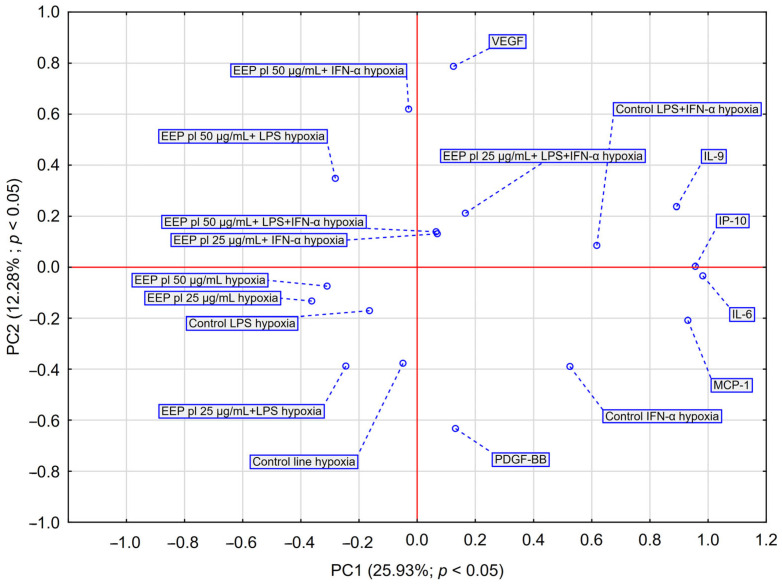
PCA score plot illustrating data regarding the impact of EEP-PL (25–50 μg/mL) on the secretion of IL-6, VEGF, PDGF-BB, MCP-1, IP-10 and IL-9 by astrocytes cell line CCF-STTG1 stimulated with LPS and/or IFN-α. Abbreviation: PC—principal component.

PCA analysis (Figure 4) showed that EEP-PL at a final concentrations of 50 μg/mL with IFN-α and EEP-PL at a final concentration of 50 μg/mL with LPS showed a similar activity on cytokine secretion by astrocytes (upper left quadrant). Similar effects were also demonstrated for EEP-PL 50 μg/mL + LPS + IFN-α, EEP-PL 25 μg/mL + IFN-α and EEP-PL 25 μg/mL + LPS + IFN-α (upper right quadrant). Similar effects on each other were also exhibited by EEP-PL 50 μg/mL, EEP-PL 25 μg/mL and EEP-PL 25 μg/mL + LPS (lower left quadrant). Of note, the sample treated with EEP 50 µg/mL + IFN-α shows the greatest shift along the PC2 axis in the PCA space among all EEP combinations. The variables with the greatest impact were IL-6, IP-10, MCP-1, and IL-9, all showing a high index (>0.85), suggesting that these cytokines play a key role in the differential response profiles to EEP-mediated stimulation in the cells studied. PCA also showed that EEP 50 µg/mL + IFN-α and EEP 25 µg/mL + LPS had the greatest coordinate magnitude in the PCA space, indicating strong separation from other conditions This suggests that these conditions may lead to a more pronounced modulation of the cytokine profile.

Ethanolic extract of Polish propolis (EEP-PL) and quercetin modulated the secretion of pro-inflammatory cytokines (IL-6, VEGF, PDGF-BB, IP-10, MCP-1 and IL-9) by CCF-STTG1 astrocytes under LPS, IFN-α or a combination thereof under hypoxic conditions, showing mainly inhibitory effects on IL-6, MCP-1 and PDGF-BB.

### 2.4. Comparative Effects of Quercetin on IL-6, VEGF, PDGF-BB, MCP-1, IP-10 and IL-9 Secretion in LPS and/or IFN-α Induced by Astrocyte CCF-STTG1 Cell Line 

**Figure 5:**
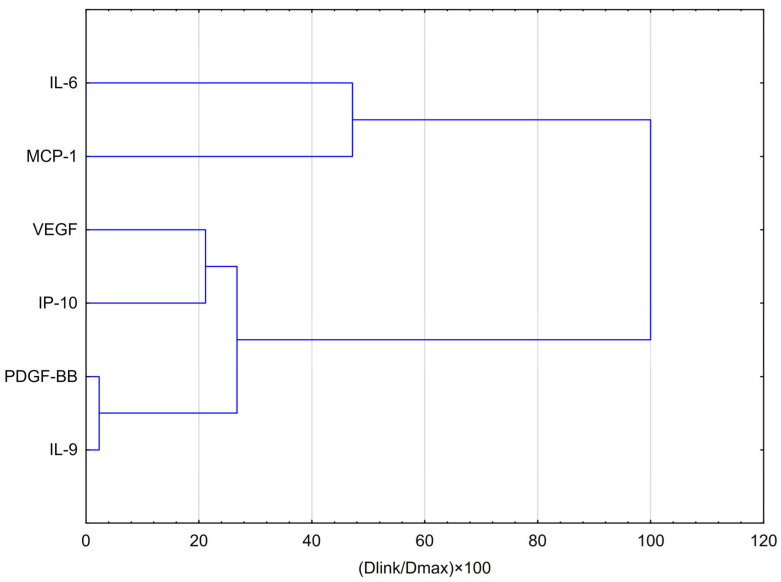
Dendrogram obtained via HCA analysis of data regarding the behaviour of IL-6, VEGF, PDGF-BB, MCP-1, IP-10 and IL-9 secretion by CCF-STTG1 cells stimulated by LPS, IFN-α and LPS + IFN-α treatment with quercetin in hypoxia conditions. Dlink denotes the linkage distance, or the measured separation between two clusters. Dmax indicates the maximum possible distance that may occur between any clusters.

HCA analysis (Figure 5) showed that the first and most compact cluster comprises DGF-BB, IL-9, and IP-10, which were grouped at the shortest distances (below 20% of Dmax). This indicates a high degree of similarity in their behaviour, suggesting similar expression patterns or potential co-regulation in response to EEP and hypoxia. A second cluster emerged with the inclusion of VEGF in the previous group, implying a moderate similarity between VEGF and the PDGF-BB/IL-9/IP-10–VEGF may exhibit a comparable, though not identical, mode of action. The third cluster consists of IL-6 and MCP-1, reflecting their shared behaviour as strongly pro-inflammatory cytokines. Their profile differs significantly from that of the other cytokines, as they clustered only at the largest relative distance (~100% of Dmax), indicating they are the least similar to the cytokines in the other clusters. Cytokines in the first cluster (PDGF-BB, IL-9, and IP-10) may be co-regulated or exhibit a similar response to EEP-PL.

**Figure 6:**
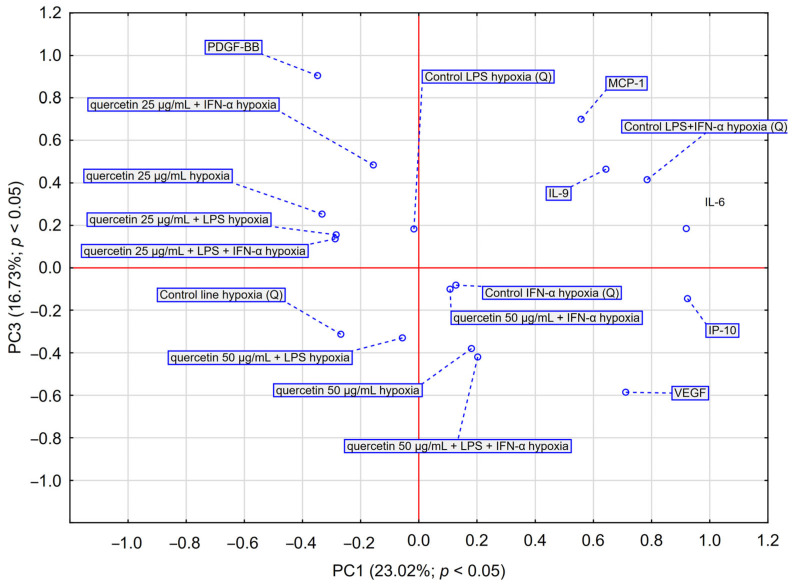
PCA score plot illustrating data regarding the impact of quercetin (25–50 μg/mL) on the secretion of IL-6, VEGF, PDGF-BB, MCP-1, IP-10 and IL-9 by astrocyte cell line CCF-STTG1 stimulated with LPS and/or IFN-α treatment with quercetin in hypoxia conditions. Abbreviation: PC—principal component.

PCA analysis (Figure 6) showed that quercetin 50 µg/mL + LPS + IFN-α and quercetin 50 µg/mL caused a shift toward VEGF and IP-10, suggesting that these conditions may have the greatest effect on the release of these cytokines by astrocytes. Quercetin at 25 µg/mL appears to have a moderate effect on cytokine expression, particularly in the presence of single stimulators (LPS or IFN-α). However, at a higher concentration (50 µg/mL) and in combination with LPS + IFN-α, a shift in the cytokine profile toward inflammatory mediators (IL-6, IP-10, VEGF) is observed, suggesting a potential immunostimulatory effect under hypoxic conditions. Taken together, the data suggest that higher quercetin concentrations, when combined with inflammatory stimulation, may result in an altered proinflammatory cytokine profile, particularly IL-6, IP-10 and VEGF, under hypoxic conditions. Quercetin exhibited a dose- and condition-dependent effect on cytokine secretion, with higher concentrations and the presence of inflammatory stimuli leading to a more pronounced reduction in IL-6, VEGF, and IP-10 levels. These findings suggest its potential immunomodulatory effects under hypoxic conditions.

## 3. Discussion

High-grade adult-type diffuse gliomas have a five-year survival rate of approximately 5%. The standard treatment typically includes surgical removal of the tumour, followed by a combination of temozolomide chemotherapy and radiation therapy [16]. These aggressive primary brain tumours are characterized by significant heterogeneity and vascular proliferation. Hypoxic conditions in the tumour microenvironment are considered a key factor contributing to tumour progression [17]. To be effective against glioma, a substance must be capable of crossing the blood–brain barrier (BBB). Flavonoids found in propolis demonstrate selective BBB permeability, including compounds such as caffeic acid phenethyl ester (CAPE) [18], pinocembrin [19], quercetin [20], naringin [21], genistein [22], and epigallocatechin or its metabolites [23]. The therapeutic potential of propolis may, in part, result from synergistic interactions among polyphenols and other bioactive constituents. To fully evaluate its efficacy, further in vivo studies are needed, particularly those examining repeated administration, compound bioavailability, and BBB permeability. Interestingly Thammasit P. et al. have shown that formulated novel poly (n-butylcyanoacrylate) nanoparticles (PBCA-NP) containing propolis pass through a BBB model with great efficiency, suggesting highly promising targeting of brain infections [24].

Neither quercetin nor propolis reduced the viability of the tested cells. According to ISO 10993-5 [25], which outlines guidelines for the biological assessment of medical devices, a cell viability of less than 70% indicates cytotoxicity [26]. In this study, Polish ethanolic extract of propolis and quercetin concentrations of 25 and 50 μg/mL maintained cell viability above this level, suggesting they are not cytotoxic.

Due to the ability of the compounds in propolis to cross the blood–brain barrier (BBB) and its known influence on tumour-related processes such as proliferation, differentiation, and apoptosis through multiple molecular targets, we used Polish propolis and one of its main components, quercetin, in our study to investigate their immunomodulatory effects on highly malignant adult-type diffuse glioma cells. Dong-Ping Sang et al. demonstrated that the combined treatment with TMZ and quercetin effectively inhibits the survival of human U251 and U87 glioma cells in vitro [27]. In contrast, the team led by Markiewicz-Żukowska R. found that both TMZ and EEP, when administered individually, exerted a dose- and time-dependent inhibitory effect on the growth of the U87MG cell line, as evidenced by a gradual decrease in cell viability and altered proliferation rates. The antitumour effect of TMZ was enhanced by EEP, particularly after short exposure times, where the simultaneous application of TMZ and EEP resulted in greater growth inhibition than either agent alone. Furthermore, prolonged treatment with TMZ did not alter NF-κB activity, while EEP alone caused only a slight reduction in nuclear translocation of this transcription factor. In contrast, combined treatment with TMZ and EEP led to an approximately twofold reduction in NF-κB activity [14].

Pro-inflammatory cytokines play a key role in tumour development and progression by modulating the tumour microenvironment, regulating the immune response and influencing angiogenesis, lymphangiogenesis and metastasis [28,29]. IL-6 is one of the most important pro-inflammatory mediators involved in cancer initiation and progression. Its actions are multifaceted and involve numerous molecular mechanisms that determine the behaviour of cancer cells. Reducing IL-6 levels has beneficial effects in cancer therapy. VEGF is one of the most potent stimulators of angiogenesis, a process essential for tumour growth and infiltration of tissue. Hypoxia within gliomas has been shown to activate hypoxia-inducible factor 1 alpha (HIF-1α), which in turn promotes tumour neoangiogenesis by stimulating VEGF expression [23]. VEGF also acts synergistically with other cytokines and growth factors to enhance tumour angiogenesis. A decrease in VEGF concentration is associated with the inhibition of angiogenesis and is therefore beneficial in cancer therapy.

In our previous studies, we demonstrated that the ethanolic extract of Brazilian green propolis may potentially inhibit tumour progression in grade IV glioblastoma, in part by reducing IL-6 and VEGF concentrations under both hypoxic and normoxic conditions [30]. In our current study, we demonstrated that Polish ethanolic extracts of propolis at final concentrations of 25 µg/mL and 50 µg/mL significantly reduced IL-6 concentrations secreted by the CCF-STTG1 astrocyte cell line. In contrast, quercetin alone did not significantly decrease IL-6 levels; a statistically significant reduction was observed only when quercetin was combined with LPS and IFN-α. Michaud-Levesque et al. demonstrated that quercetin is a potent inhibitor of the IL-6-induced JAK/STAT3 signalling pathway. Quercetin significantly reduces T98G and U87 glioma cell proliferation and migration by inhibiting STAT3 phosphorylation, primarily through the downregulation of two STAT3 target genes: *cyclin D1* and *MMP-2*. Moreover, STAT3 binds to the *cyclin D1* promoter in the presence of IL-6, and quercetin antagonizes this recruitment [31].

In our study, we showed that, that on the other hand, quercetin at a final concentration of 25 µg/mL reduced VEGF levels. Propolis contains a variety of compounds, including flavonoids, which may not always produce the same effects in combination as they do individually. We noticed that EEP PL at concentration of 25 µg/mL decreases the concentration of VEGF in the model with LPS. It is worth highlighting that VEGF acts synergistically with other cytokines and growth factors like PDGF-BB in promoting tumour angiogenesis. PDGF-BB is a member of the PDGF family, which plays an important role in cancer progression and metastasis. The angiogenic activity of PDGF-BB can be enhanced in the presence of other angiogenic factors, which indicates a synergistic action of growth factors in the angiogenesis process of cancer [32]. Therefore, reduction of the PDGF-BB concentration seems to be beneficial for oncology patients. In our experiment, a significant reduction was observed with EEP-PL at a concentration of 50 µg/mL following INF-α stimulation, as well as with quercetin at the same concentration following LPS and INF-α stimulation. It is known that PDGF-BB inhibitors can suppress tumour cell growth, angiogenesis, and lymphangiogenesis, which may effectively impede tumour progression and tissue infiltration. Therefore, EEP-PL and quercetin may be considered potential inhibitors of PDGF-BB. However, our observations should be confirmed in future studies.

Another cytokine of importance in carcinogenesis is the chemokine IP-10 (CXCL10), which is associated with chronic inflammation, immune dysfunction, and cancer development [33]. Therefore, decreasing the concentration of IP-10 in the microenvironment of the tumour seems to be beneficial for oncology patients. Maru S. have shown that stimulation by IP-10 (CXCL10) enhances ERK1/2 activation, promoting the proliferation of glioblastoma multiforme cells [34]. In our experiment, propolis at both concentrations significantly decreased CXCL10 levels following IFN-α and LPS + IFN-α stimulation, with a stronger effect observed at the higher concentration. This suggests that the therapeutic effect is dose-dependent. In contrast, quercetin was significantly more effective at reducing IP-10 levels at lower concentrations (Figure 2h). In addition to IP-10, we also examined the effects of propolis and quercetin on another chemokine, MCP-1 (CCL2), which is involved in macrophage recruitment and the initiation of inflammation—processes that play a key role in the tumour microenvironment [35]. In the case of MCP-1, a decrease in concentration is expected due to its key roles in tumour progression, cell proliferation and invasiveness. Additionally, it strongly creates the tumour microenvironment.

The main receptor for CCL2 is CCR2. Studies on the CCL2–CCR2 axis have shown that it plays multiple roles in tumour progression, including promoting tumour cell proliferation and invasiveness, as well as shaping the tumour microenvironment by enhancing angiogenesis and recruiting immunosuppressive cells [36]. EEP-PL, at both concentrations, significantly reduced MCP-1 levels across all models. In contrast, quercetin showed a less pronounced effect on MCP-1 reduction.

IL-9 is a cytokine with strong pro-inflammatory properties. Pro-inflammatory cytokines play a crucial role in cancer pathogenesis and progression by modulating the tumour microenvironment, regulating the immune response, and influencing angiogenesis, lymphangiogenesis, and metastasis [37]. Therefore, it appears that a reduction in this cytokine would be expected during therapy. However, in our study, we did not observe a significant reduction in IL-9 levels.

The main limitation of this study was the relatively short 24-h incubation period with the test compounds. In future experiments, we plan to extend the incubation time and reassess cytotoxicity under those conditions. In summary, our study highlights that IL-6, VEGF, PDGF-BB, IP-10, MCP-1, and IL-9 exhibit complex and often context-dependent roles in tumours, making them both potential biomarkers and therapeutic targets. Despite extensive research, only a limited number of FDA-approved drugs are currently available for glioma treatment, and their effectiveness varies among patients. Polish propolis and quercetin were shown to influence the concentrations of the aforementioned cytokines and may therefore be considered promising natural agents to support the treatment of grade IV glioma through immunomodulatory effects. However, a limitation of this study is the lack of in vivo validation. Further in vivo studies and clinical trials are necessary to confirm the therapeutic potential of propolis and its active compound, quercetin.

## 4. Materials and Methods

### 4.1. Preparation of Polish EEP

Samples of Polish propolis were obtained from an apiary in Kamianna, Poland. Raw propolis was manually harvested from beehives and dried before further processing. The dried sample was then extracted with 70% ethanol (*v*/*v*) in a three-step process. The ratio of crude propolis to ethanol was 1:10. In the first stage, a mixture of propolis and ethanol was stirred for 24 h. Next, the extraction mixture was filtered under a vacuum through the paper filter. In the second stage, the sediment was mixed again with 70% ethanol and stirred for 24 h. After this, the filtration was repeated. In third stage, the filtrate was added to the previous filtrate, and the sediment was finally extracted with 70% ethanol as described Kurek-Górecka et al. [12]. Finally, the last extract was added to previously obtained extracts. The joined extracts were solid in the vacuum at 40 °C. Firstly, the extract was concentrated to the consistency of thick syrup on a rotary vacuum evaporator (IKA-Werke RV 05 Basic, Staufen, Germany). Then, the obtained joined extract was evaporated to dryness in a vacuum oven (Infitek DOV-25H, Lijian, China). Finally, the EEP PL was pre-dissolved in DMSO purchased from Sigma Chemical Company (St. Louis, MO, USA) and then dissolved in culture medium to a final concentration of 25 and 50 µg/mL (the final concentration of DMSO was 0.1%). The phenolic compounds present in the extract were previously identified and quantified using HPLC analysis [15].

### 4.2. Cell Culture of Astrocyte Cell Line CCF-STTG1

The experiments were carried out using the CCF-STTG1 astrocyte cell line, obtained from the American Type Culture Collection (ATCC, Manassas, VA, USA). This cell line was originally derived from the brain tissue of a 68-year-old Caucasian woman diagnosed with grade IV astrocytoma. The CCF-STTG1 cells were cultured at 37 °C in monolayers using RPMI 1640 medium supplemented with 10% foetal bovine serum (FBS) (ATCC, Manassas, VA, USA), 100 U/mL penicillin, and 100 μg/mL streptomycin from PAA Laboratories (Pasching, Austria). The cells were maintained in a humidified incubator at 37 °C with an atmosphere of 5% CO_2_ and 95% air. Cell passages were performed twice weekly, and for experimental procedures, cell suspensions at a concentration of 5 × 10^4^ cells/mL were used.

### 4.3. Astrocyte Cell Line CCF-STTG1 Stimulation with Polish Ethanolic Extracts of Propolis or Quercetin with LPS, IFN-α, and Their Combination (LPS + IFN-α)

In the next phase of the study, cells were seeded into 96-well plates at a volume of 200 µL per well and incubated for 48 h to allow for adhesion. Following this, the cells were treated with Polish ethanolic extracts of propolis at final concentrations of 25 and 50 μg/mL, and with quercetin obtained from Sigma-Aldrich (Darmstadt, Germany) at the same concentrations (pre-dissolved in DMSO and then dissolved in culture medium—the final concentration of DMSO was 0.1%), either alone or in combination with LPS (200 ng/mL) and/or IFN-α (100 U/mL). Lipopolysaccharide BE. Coll 026:B6 was purchased from Difco Laboratories (Detroit, MI, USA). Interferon-alpha (IFN-α) 3 MIU/0.5 mL was purchased from Schering-Plough, Brinny, Ireland. Immediately after adding the compounds, the plate was placed in a hypoxic incubator with 1% oxygen for 2 h. After the hypoxic exposure, the plate was returned to standard culture conditions (5% CO_2_ and 95% air) for the remaining 22 h. All experiments were performed in triplicate.

### 4.4. MTT Assay

Cytotoxicity was assessed using the MTT (St. Louis, MO, USA) assay (3-(4,5-dimethylthiazol-2-yl)-2,5-diphenyltetrazolium bromide), following previously established methods [38]. Quercetin was applied to 96-well plates at final concentrations of 25 and 50 μg/mL, either alone or in combination with LPS, IFN-α, or both LPS and IFN-α. Each well contained a total volume of 200 μL. After 24 h of incubation, the medium was collected and stored at −80 °C for later cytokine analysis. Subsequently, 180 μL of fresh medium and 20 μL of MTT solution (5 mg/mL in PBS) were added to each well and incubated for 4 h. After incubation, the supernatants were discarded, and DMSO was added to solubilise the formazan crystals. Absorbance was then measured at 550 nm using an EonTM Microplate Spectrophotometer (BioTek, Winooski, VT, USA).

### 4.5. xMAP Luminex Multiplex Immunoassay

The concentrations of the cytokines under investigation were measured using the Luminex Multiplex Immunoassay. This method uses sets of magnetic beads, each coded with different shades of red and coated with antibodies specific to the target cytokines. When these beads are mixed with sample supernatants and standards, they bind to their corresponding analytes. Detection is achieved by adding biotinylated antibodies, which then interact with a streptavidin–-phycoerythrin conjugate.

Between incubation steps, the ferromagnetic beads were washed using an ELx 50 magnetic washer (Bio-Tek, Winooski, VT, USA). Quantitative analysis was carried out using the xMAP Luminex Multiplex Immunoassay system, along with the Multiplex Bead-Based Cytokine Kit (Bio-Plex 3D Suspension Array System and Luminex xPonent Software, Version 4.3, Bio-Rad). We used 27-plex Bio-Plex Pro Human Cytokine Grp/Panel 27-Plex cat #M500KCAF0Y BIO-RAD Laboratories, Hercules, CA, USA. This system relies on a flow cytometry-based approach with two lasers—one (638 nm) to identify the bead colour corresponding to a specific analyte and the other (532 nm) to excite phycoerythrin, producing a fluorescence signal proportional to the analyte concentration. Final cytokine concentrations were calculated using standard curves generated from known reference values.

### 4.6. Statistical Analysis

All experiments were performed in triplicate, and the results are presented as mean values. Statistical analyses were carried out using STATISTICA 13.1 software (StatSoft Inc., Tulsa, OK, USA). Data evaluation involved hierarchical cluster analysis (HCA) using full linkage and Euclidean distance, as well as principal component analysis (PCA). The PCA model was developed using the NIPALS iterative algorithm, with a convergence criterion of 0.00001 and a maximum of 50 iterations. The number of components was selected based on optimal predictive performance determined through repeated cross-validations, with an upper limit imposed. The final PCA model was reduced to two components. The PCA analysis, represented in a PC1 vs. PC2 loadings plot, identified the variables contributing most significantly to dataset variability and revealed key correlations among them. PCA and HCA were employed to detect natural groupings within the data and to evaluate the distinct effects of EEP-PL and quercetin on astrocytes. A one-way ANOVA was used to compare the effects of EEP-PL and quercetin at different concentrations on cytokine production induced by LPS, IFN-α, and their combination. The MTT assay results were analysed using a *t*-test. A *p*-value below 0.05 was considered statistically significant.

## 5. Conclusions

In our study, we showed that Polish propolis and its compound, quercetin, affect the tumour microenvironment in vitro, in particular by influencing the concentrations of selected cytokines. Glioblastoma and IDH-mutant grade 4 astrocytoma are the most prevalent forms of high-grade adult-type diffuse gliomas. Standard treatment for diffuse gliomas typically involves surgical removal of the tumour, followed by a combination of temozolomide chemotherapy and radiation therapy. However, tissue hypoxia increases resistance to treatment. Hypoxia-inducible factor 1 plays a key role in the reprogramming of cancer metabolism. Therefore, conducting studies under hypoxic conditions is particularly important. IL-6, VEGF, PDGF-BB, IP-10, MCP-1, and IL-9 exhibit complex, often context-dependent functions in tumours, making them potential biomarkers and therapeutic targets. Polish propolis and quercetin were shown to modulate the concentrations of these cytokines and may therefore be considered promising natural agents to support astrocytoma therapy through immunomodulatory effects. In particular, the significant reduction in concentrations of IL-6, MCP-1, PDGF-BB by Polish propolis and quercetin seems to be important in cancer therapy. Future studies are required because the role of the assessed cytokines in tumour progression, cell proliferation and invasiveness is crucial.

## Data Availability

Data are contained within the article.

## Supplementary Materials

The following supporting information can be downloaded at: https://www.mdpi.com/article/10.3390/molecules30143008/s1, Table S1. Effect of Ethanolic Extract of Propolis (EEP) and Quercetin on the Levels (pg/mL) of Inflammatory Cytokines (IL-6, VEGF, PDGF-BB, IP-10, MCP-1, IL-9) in Glioma Cells under Hypoxic Conditions—Mean Values, Standard Deviations (SD), and Coefficients of Variation (%CV). Table S2. Statistical Comparison of IL-6 Levels between Experimental Groups Treated with Ethanolic Extract of Propolis (EEP) in Glioma Cells under Hypoxic Conditions (LSD Post Hoc Test). Table S3. Statistical Comparison of VEGF Levels between Experimental Groups Treated with Ethanolic Extract of Propolis (EEP) in Glioma Cells under Hypoxic Conditions (LSD Post Hoc Test). Table S4. Statistical Comparison of PDGF-BB Levels between Experimental Groups Treated with Ethanolic Extract of Propolis (EEP) in Glioma Cells under Hypoxic Conditions (LSD Post Hoc Test). Table S5. Statistical Comparison of IL-10 Levels between Experimental Groups Treated with Ethanolic Extract of Propolis (EEP) in Glioma Cells under Hypoxic Conditions (LSD Post Hoc Test). Table S6. Statistical Comparison of MCP-1 Levels between Experimental Groups Treated with Ethanolic Extract of Propolis (EEP) in Glioma Cells under Hypoxic Conditions (LSD Post Hoc Test). Table S7. Statistical Comparison of IL-9 Levels between Experimental Groups Treated with Ethanolic Extract of Propolis (EEP) in Glioma Cells under Hypoxic Conditions (LSD Post Hoc Test). Table S8. Multivariate Analysis of the Effect of Ethanolic Extract of Propolis (EEP) on Cytokine Expression in Glioma Cells under Hypoxic Conditions. Table S9. Statistical Comparison of IL-6 Levels between Experimental Groups Treated with Quercetin in Glioma Cells under Hypoxic Conditions (LSD Post Hoc Test). Table S10. Statistical Comparison of VEGF Levels between Experimental Groups Treated with Quercetin in Glioma Cells under Hypoxic Conditions (LSD Post Hoc Test). Table S11. Statistical Comparison of PDGF-BB Levels between Experimental Groups Treated with Quercetin in Glioma Cells under Hypoxic Conditions (LSD Post Hoc Test). Table S12. Statistical Comparison of IL-10 Levels between Experimental Groups Treated with Quercetin in Glioma Cells under Hypoxic Conditions (LSD Post Hoc Test). Table S13. Statistical Comparison of MCP-1 Levels between Experimental Groups Treated with Quercetin in Glioma Cells under Hypoxic Conditions (LSD Post Hoc Test). Table S14. Statistical Comparison of IL-9 Levels between Experimental Groups Treated with Quercetin in Glioma Cells under Hypoxic Conditions (LSD Post Hoc Test). Table S15. Multivariate Analysis of the Quercetin on Cytokine Expression in Glioma Cells under Hypoxic Conditions.

## Abbreviations

The following abbreviations are used in this manuscript:

CNS	Central Nervous System
VEGF	Vascular Endothelial Growth Factor
HIF	Hypoxia-Inducible Factor
IP-10	Interferon Gamma-Induced Protein 10
PDGF-BB	Platelet-Derived Growth Factor
MCP-1	Monocyte Chemoattractant Protein-1
WHO	World Health Organization
LPS	Lipopolysaccharide
