# Supplementary material for: The Potential Anti-Cancer Effects of Polish Ethanolic Extract of Propolis and Quercetin on Glioma Cells Under Hypoxic Conditions
Source: Molecules. 2025 Jul 17;30(14):3008. doi: 10.3390/molecules30143008 (PMC12298029; doi:10.3390/molecules30143008)
Supplement: Supplementary file 1 [file molecules-30-03008-s001.zip › molecules-3703678-supplementary.pdf]

Article

The Potential Anti-Cancer Effect of Polish Ethanolic Extract of Propolis and Quercetin on Glioma Cells Under Hypoxic Condition

Małgorzata Kłósek[1]*, Anna Kurek-Górecka[1]*, Radosław Balwierz[2], Grażyna Pietsz[1], Zenon P. Czuba[1]

[1] Department of Microbiology and Immunology, Faculty of Medical Sciences, Medical University of Silesia in Katowice, Jordana 19. 41-808 Zabrze, Poland; mklosek@sum.edu.pl (M.K.); akurekgorecka@sum.edu.pl (A.K.-G.); gpietsz@sum.edu.pl (G.P.); zczuba@sum.edu.pl (Z.P.C.)

[2] Institute of Chemistry, University of Opole, Oleska 48, 45-052 Opole, Poland; radoslaw.balwierz@uni.opole.pl

* Correspondence: mklosek@sum.edu.pl; akurekgorecka@sum.edu.pl; Tel./Fax: +48-322-722-554

Content

Table S1. Effect of Ethanolic Extract of Propolis (EEP) and Quercetin on the Levels (pg/mL) of Inflammatory Cytokines (IL-6, VEGF, PDGF-BB, IP-10, MCP-1, IL-9) in Glioma Cells under Hypoxic Conditions – Mean Values, Standard Deviations (SD), and Coefficients of Variation (%CV).

Table S2. Statistical Comparison of IL-6 Levels between Experimental Groups Treated with Ethanolic Extract of Propolis (EEP) in Glioma Cells under Hypoxic Conditions (LSD Post Hoc Test).

Table S3. Statistical Comparison of VEGF Levels between Experimental Groups Treated with Ethanolic Extract of Propolis (EEP) in Glioma Cells under Hypoxic Conditions (LSD Post Hoc Test).

Table S4. Statistical Comparison of PDGF-BB Levels between Experimental Groups Treated with Ethanolic Extract of Propolis (EEP) in Glioma Cells under Hypoxic Conditions (LSD Post Hoc Test).

Table S5. Statistical Comparison of IL-10 Levels between Experimental Groups Treated with Ethanolic Extract of Propolis (EEP) in Glioma Cells under Hypoxic Conditions (LSD Post Hoc Test).

Table S6. Statistical Comparison of MCP-1 Levels between Experimental Groups Treated with Ethanolic Extract of Propolis (EEP) in Glioma Cells under Hypoxic Conditions (LSD Post Hoc Test).

Table S7. Statistical Comparison of IL-9 Levels between Experimental Groups Treated with Ethanolic Extract of Propolis (EEP) in Glioma Cells under Hypoxic Conditions (LSD Post Hoc Test).

Table S8. Multivariate Analysis of the Effect of Ethanolic Extract of Propolis (EEP) on Cytokine Expression in Glioma Cells under Hypoxic Conditions.

Table S9. Statistical Comparison of IL-6 Levels between Experimental Groups Treated with Quercetin in Glioma Cells under Hypoxic Conditions (LSD Post Hoc Test).

Table S10. Statistical Comparison of VEGF Levels between Experimental Groups Treated with Quercetin in Glioma Cells under Hypoxic Conditions (LSD Post Hoc Test).

Table S11. Statistical Comparison of PDGF-BB Levels between Experimental Groups Treated with Quercetin in Glioma Cells under Hypoxic Conditions (LSD Post Hoc Test).

Table S12. Statistical Comparison of IL-10 Levels between Experimental Groups Treated with Quercetin in Glioma Cells under Hypoxic Conditions (LSD Post Hoc Test).

Table S13. Statistical Comparison of MCP-1 Levels between Experimental Groups Treated with Quercetin in Glioma Cells under Hypoxic Conditions (LSD Post Hoc Test).

Table S14. Statistical Comparison of IL-9 Levels between Experimental Groups Treated with Quercetin in Glioma Cells under Hypoxic Conditions (LSD Post Hoc Test).

Table S15. Multivariate Analysis of the Quercetin on Cytokine Expression in Glioma Cells under Hypoxic Conditions.

Table S1. Effect of Ethanolic Extract of Propolis (EEP) and Quercetin on the Levels (pg/mL) of Inflammatory Cytokines (IL-6, VEGF, PDGF-BB, IP-10, MCP-1, IL-9) in Glioma Cells under Hypoxic Conditions – Mean Values, Standard Deviations (SD), and Coefficients of Variation (%CV)

Sample	IL-6			VEGF			PDGF-BB			IP-10			MCP-1			IL-9		
	Value	SD	%CV	Value	SD	%CV	Value	SD	%CV	Value	SD	%CV	Value	SD	%CV	Value	SD	%CV
Control line	872.09	30.41	3.49	136.51	21.95	16.08	7.82	5.71	73.01	4.09	0.82	20.00	804.44	28.33	3.52	21.71	0.99	4.55
Control LPS	749.65	53.45	7.13	141.20	6.17	4.37	4.15	3.74	90.08	5.18	1.38	26.70	797.19	9.45	1.19	20.41	3.57	17.48
Control IFN-α	1236.63	106.30	8.60	151.98	17.67	11.63	9.29	7.02	75.59	162.57	16.85	10.37	1348.15	22.46	1.67	23.86	4.53	18.99
Control LPS+IFN-α	1259.34	157.66	12.52	172.29	11.69	6.79	4.71	2.61	55.38	182.59	15.42	8.45	1304.77	127.14	9.74	25.80	2.60	10.09
EEP pl 25 μg/mL	611.33	61.79	10.11	134.90	9.47	7.02	3.12	1.95	62.55	4.09	0.82	20.00	438.28	12.81	2.92	19.12	1.31	6.85
EEP pl 50 μg/mL	597.75	53.42	8.94	203.38	35.04	17.23	10.94	2.26	20.68	3.19	1.44	45.18	289.97	30.82	10.63	20.09	2.08	10.35
EEP pl 25 μg/mL+LPS	612.87	48.71	7.95	97.57	45.61	46.75	4.72	3.37	71.42	3.46	1.65	47.76	496.26	39.13	7.89	21.82	1.17	5.34
EEP pl 50 μg/mL+ LPS	638.85	27.41	4.29	188.51	16.39	8.69	2.51	2.66	106.08	2.73	1.99	73.03	321.78	45.39	14.11	21.17	3.18	15.04
EEP pl 25 μg/mL+ IFN-α	844.66	77.33	9.16	168.62	8.64	5.13	2.44	2.68	109.88	83.89	10.78	12.84	947.17	73.85	7.80	21.82	0.86	3.92
EEP pl 50 μg/mL+ IFN-α	841.66	272.83	32.42	209.47	2.91	1.39	0.58	0.82	140.61	33.01	10.80	32.72	556.20	70.63	12.70	23.10	4.37	18.91
EEP pl 25 μg/mL+ LPS+IFN-α	884.25	155.86	17.63	174.15	11.46	6.58	2.90	4.83	166.63	73.50	17.03	23.16	959.04	100.59	10.49	23.97	3.54	14.75
EEP pl 50 μg/mL+ LPS+IFN-α	923.41	107.59	11.65	193.98	7.99	4.12	6.94	8.88	127.95	37.81	3.89	10.30	651.55	81.03	12.44	23.43	1.12	4.77
Control line (Q)	991.38	116.40	11.74	300.53	27.34	9.10	7.22	4.97	68.93	19.70	5.44	27.61	1229.47	125.19	10.18	24.72	0.64	2.61
Control LPS (Q)	1373.01	151.71	11.05	269.13	15.28	5.68	10.18	7.22	70.96	28.15	8.01	28.44	1452.33	300.98	20.72	33.29	3.53	10.60

Control IFN-α (Q)	1510.39	287.94	19.06	277.02	23.60	8.52	6.40	1.79	27.95	430.32	113.21	26.31	1335.49	3.87	0.29	31.79	3.90	12.26
Control LPS+IFN-α (Q)	2683.45	1093.40	40.75	359.55	94.48	26.28	12.82	5.29	41.27	709.03	95.78	13.51	1699.70	141.53	8.33	39.15	3.05	7.78
quercetin 25 μg/mL	693.90	201.17	28.99	223.59	45.51	20.35	17.71	1.88	10.61	12.67	2.70	21.32	1294.52	79.57	6.15	31.81	6.68	21.00
quercetin 25 μg/mL + LPS	786.33	113.48	14.43	241.30	37.90	15.71	15.54	1.87	12.06	8.33	2.64	31.66	1241.20	74.59	6.01	32.96	2.10	6.37
quercetin 25 μg/mL + IFN-α	798.57	171.48	21.47	249.34	24.99	10.02	19.89	5.65	28.41	23.32	3.27	14.05	1481.19	155.33	10.49	35.22	4.70	13.34
quercetin 25 μg/mL + LPS + IFN-α	779.40	29.65	3.80	238.74	3.25	1.36	14.46	3.74	25.87	7.32	3.50	47.86	1271.12	37.91	2.98	31.98	2.63	8.23
quercetin 50 μg/mL	1284.03	172.74	13.45	352.31	41.35	11.74	2.27	1.68	74.19	397.52	43.65	10.98	1217.92	129.11	10.60	33.58	4.49	13.37
quercetin 50 μg/mL + LPS	824.21	80.73	9.79	334.70	28.01	8.37	3.91	3.31	84.75	215.34	13.10	6.09	1350.90	45.32	3.36	27.62	3.90	14.12
quercetin 50 μg/mL + IFN-α	1022.86	81.36	7.95	291.19	16.13	5.54	7.06	8.57	121.38	451.03	44.75	9.92	1257.19	60.71	4.83	35.96	3.31	9.19
quercetin 50 μg/mL + LPS + IFN-α	1206.23	135.63	11.24	403.93	30.68	7.59	3.83	5.70	148.81	300.84	13.95	4.64	1173.78	17.06	1.45	35.53	2.58	7.25

Table S2. Statistical Comparison of IL-6 Levels between Experimental Groups Treated with Ethanolic Extract of Propolis (EEP) in Glioma Cells under Hypoxic Conditions (LSD Post Hoc Test)

	LSD test; variable IL-6<br>Probabilities for Post Hoc Tests<br>Error: Between MS = 13835.. df = 24.000												
Cell No.	Sample	{1}	{2}	{3}	{4}	{5}	{6}	{7}	{8}	{9}	{10}	{11}	{12}
1	Control line hypoxia		0.2146	0.0009	0.0005	0.0121	0.0087	0.0125	0.0230	0.7776	0.7541	0.9003	0.5980
2	Control LPS hypoxia	0.2146		0.0000	0.0000	0.1627	0.1268	0.1673	0.2600	0.3324	0.3476	0.1739	0.0829
3	Control IFN-α hypoxia	0.0009	0.0000		0.8151	0.0000	0.0000	0.0000	0.0000	0.0004	0.0004	0.0012	0.0033
4	Control LPS+IFN-α hypoxia	0.0005	0.0000	0.8151		0.0000	0.0000	0.0000	0.0000	0.0002	0.0002	0.0007	0.0019
5	EEP pl 25 µg/mL hypoxia	0.0121	0.1627	0.0000	0.0000		0.8888	0.9873	0.7769	0.0230	0.0246	0.0090	0.0034
6	EEP pl 50 µg/mL hypoxia	0.0087	0.1268	0.0000	0.0000	0.8888		0.8762	0.6725	0.0168	0.0180	0.0065	0.0024
7	EEP pl 25 µg/mL+ LPS hypoxia	0.0125	0.1673	0.0000	0.0000	0.9873	0.8762		0.7891	0.0238	0.0255	0.0094	0.0035
8	EEP pl 50 µg/mL+ LPS hypoxia	0.0230	0.2600	0.0000	0.0000	0.7769	0.6725	0.7891		0.0425	0.0453	0.0174	0.0068
9	EEP pl 25 µg/mL+ IFN-α hypoxia	0.7776	0.3324	0.0004	0.0002	0.0230	0.0168	0.0238	0.0425		0.9753	0.6838	0.4203
10	EEP pl 50 µg/mL+ IFN-α hypoxia	0.7541	0.3476	0.0004	0.0002	0.0246	0.0180	0.0255	0.0453	0.9753		0.6614	0.4030
11	EEP pl 25 µg/mL+ LPS+IFN-α hypoxia	0.9003	0.1739	0.0012	0.0007	0.0090	0.0065	0.0094	0.0174	0.6838	0.6614		0.6870
12	EEP pl 50 µg/mL+ LPS+IFN-α hypoxia	0.5980	0.0829	0.0033	0.0019	0.0034	0.0024	0.0035	0.0068	0.4203	0.4030	0.6870	

Table S3. Statistical Comparison of VEGF Levels between Experimental Groups Treated with Ethanolic Extract of Propolis (EEP) in Glioma Cells under Hypoxic Conditions (LSD Post Hoc Test)

	LSD test; variable VEGF<br>Probabilities for Post Hoc Tests<br>Error: Between MS = 409.47. df = 24.000												
Cell No.	Sample	{1}	{2}	{3}	{4}	{5}	{6}	{7}	{8}	{9}	{10}	{11}	{12}
1	Control line hypoxia		0.7789	0.3585	0.0405	0.9232	0.0005	0.0269	0.0044	0.0638	0.0002	0.0319	0.0019
2	Control LPS hypoxia	0.7789		0.5204	0.0720	0.7063	0.0010	0.0143	0.0086	0.1100	0.0004	0.0576	0.0039
3	Control IFN-α hypoxia	0.3585	0.5204		0.2307	0.3116	0.0048	0.0031	0.0368	0.3238	0.0019	0.1920	0.0179
4	Control LPS+IFN-α hypoxia	0.0405	0.0720	0.2307		0.0329	0.0721	0.0001	0.3361	0.8259	0.0339	0.9113	0.2017
5	EEP pl 25 µg/mL hypoxia	0.9232	0.7063	0.3116	0.0329		0.0004	0.0332	0.0034	0.0524	0.0001	0.0258	0.0015
6	EEP pl 50 µg/mL hypoxia	0.0005	0.0010	0.0048	0.0721	0.0004		0.0000	0.3770	0.0460	0.7160	0.0896	0.5746
7	EEP pl 25 µg/mL+ LPS hypoxia	0.0269	0.0143	0.0031	0.0001	0.0332	0.0000		0.0000	0.0002	0.0000	0.0001	0.0000
8	EEP pl 50 µg/mL+ LPS hypoxia	0.0044	0.0086	0.0368	0.3361	0.0034	0.3770	0.0000		0.2403	0.2169	0.3934	0.7435
9	EEP pl 25 µg/mL+ IFN-α hypoxia	0.0638	0.1100	0.3238	0.8259	0.0524	0.0460	0.0002	0.2403		0.0209	0.7406	0.1378
10	EEP pl 50 µg/mL+ IFN-α hypoxia	0.0002	0.0004	0.0019	0.0339	0.0001	0.7160	0.0000	0.2169	0.0209		0.0430	0.3581
11	EEP pl 25 µg/mL+ LPS+IFN-α hypoxia	0.0319	0.0576	0.1920	0.9113	0.0258	0.0896	0.0001	0.3934	0.7406	0.0430		0.2418
12	EEP pl 50 µg/mL+ LPS+IFN-α hypoxia	0.0019	0.0039	0.0179	0.2017	0.0015	0.5746	0.0000	0.7435	0.1378	0.3581	0.2418	

Table S4. Statistical Comparison of PDGF-BB Levels between Experimental Groups Treated with Ethanolic Extract of Propolis (EEP) in Glioma Cells under Hypoxic Conditions (LSD Post Hoc Test)

	LSD test; variable PDGF-BB<br>Probabilities for Post Hoc Tests<br>Error: Between MS = 19.999. df = 24.000												
Cell No.	Sample	{1}	{2}	{3}	{4}	{5}	{6}	{7}	{8}	{9}	{10}	{11}	{12}
1	Control line hypoxia		0.3248	0.6909	0.4033	0.2100	0.4014	0.4039	0.1585	0.1535	0.0591	0.1900	0.8110
2	Control LPS hypoxia	0.3248		0.1721	0.8786	0.7797	0.0753	0.8777	0.6567	0.6435	0.3384	0.7341	0.4527
3	Control IFN-α hypoxia	0.6909	0.1721		0.2222	0.1039	0.6554	0.2226	0.0755	0.0728	0.0254	0.0927	0.5255
4	Control LPS+IFN-α hypoxia	0.4033	0.8786	0.2222		0.6657	0.1011	0.9991	0.5512	0.5390	0.2692	0.6230	0.5483
5	EEP pl 25 µg/mL hypoxia	0.2100	0.7797	0.1039	0.6657		0.0425	0.6649	0.8686	0.8542	0.4945	0.9521	0.3059
6	EEP pl 50 µg/mL hypoxia	0.4014	0.0753	0.6554	0.1011	0.0425		0.1013	0.0298	0.0287	0.0091	0.0374	0.2839
7	EEP pl 25 µg/mL+ LPS hypoxia	0.4039	0.8777	0.2226	0.9991	0.6649	0.1013		0.5504	0.5383	0.2687	0.6222	0.5490
8	EEP pl 50 µg/mL+ LPS hypoxia	0.1585	0.6567	0.0755	0.5512	0.8686	0.0298	0.5504		0.9853	0.6033	0.9161	0.2368
9	EEP pl 25 µg/mL+ IFN-α hypoxia	0.1535	0.6435	0.0728	0.5390	0.8542	0.0287	0.5383	0.9853		0.6161	0.9015	0.2298
10	EEP pl 50 µg/mL+ IFN-α hypoxia	0.0591	0.3384	0.0254	0.2692	0.4945	0.0091	0.2687	0.6033	0.6161		0.5327	0.0947
11	EEP pl 25 µg/mL+ LPS+IFN-α hypoxia	0.1900	0.7341	0.0927	0.6230	0.9521	0.0374	0.6222	0.9161	0.9015	0.5327		0.2793
12	EEP pl 50 µg/mL+ LPS+IFN-α hypoxia	0.8110	0.4527	0.5255	0.5483	0.3059	0.2839	0.5490	0.2368	0.2298	0.0947	0.2793	

Table S5. Statistical Comparison of IL-10 Levels between Experimental Groups Treated with Ethanolic Extract of Propolis (EEP) in Glioma Cells under Hypoxic Conditions (LSD Post Hoc Test)

	LSD test; variable IP-10<br>Probabilities for Post Hoc Tests<br>Error: Between MS = 89.306. df = 24.000												
Cell No.	Sample	{1}	{2}	{3}	{4}	{5}	{6}	{7}	{8}	{9}	{10}	{11}	{12}
1	Control line hypoxia		0.8892	0.0000	0.0000	1.0000	0.9077	0.9360	0.8617	0.0000	0.0010	0.0000	0.0002
2	Control LPS hypoxia	0.8892		0.0000	0.0000	0.8892	0.7986	0.8262	0.7541	0.0000	0.0014	0.0000	0.0003
3	Control IFN-α hypoxia	0.0000	0.0000		0.0159	0.0000	0.0000	0.0000	0.0000	0.0000	0.0000	0.0000	0.0000
4	Control LPS+IFN-α hypoxia	0.0000	0.0000	0.0159		0.0000	0.0000	0.0000	0.0000	0.0000	0.0000	0.0000	0.0000
5	EEP pl 25 µg/mL hypoxia	1.0000	0.8892	0.0000	0.0000		0.9077	0.9360	0.8617	0.0000	0.0010	0.0000	0.0002
6	EEP pl 50 µg/mL hypoxia	0.9077	0.7986	0.0000	0.0000	0.9077		0.9716	0.9535	0.0000	0.0007	0.0000	0.0002
7	EEP pl 25 µg/mL+ LPS hypoxia	0.9360	0.8262	0.0000	0.0000	0.9360	0.9716		0.9252	0.0000	0.0008	0.0000	0.0002
8	EEP pl 50 µg/mL+ LPS hypoxia	0.8617	0.7541	0.0000	0.0000	0.8617	0.9535	0.9252		0.0000	0.0006	0.0000	0.0001
9	EEP pl 25 µg/mL+ IFN-α hypoxia	0.0000	0.0000	0.0000	0.0000	0.0000	0.0000	0.0000	0.0000		0.0000	0.1908	0.0000
10	EEP pl 50 µg/mL+ IFN-α hypoxia	0.0010	0.0014	0.0000	0.0000	0.0010	0.0007	0.0008	0.0006	0.0000		0.0000	0.5400
11	EEP pl 25 µg/mL+ LPS+IFN-α hypoxia	0.0000	0.0000	0.0000	0.0000	0.0000	0.0000	0.0000	0.0000	0.1908	0.0000		0.0001
12	EEP pl 50 µg/mL+ LPS+IFN-α hypoxia	0.0002	0.0003	0.0000	0.0000	0.0002	0.0002	0.0002	0.0001	0.0000	0.5400	0.0001	

Table S6. Statistical Comparison of MCP-1 Levels between Experimental Groups Treated with Ethanolic Extract of Propolis (EEP) in Glioma Cells under Hypoxic Conditions (LSD Post Hoc Test)

	LSD test; variable MCP-1<br>Probabilities for Post Hoc Tests<br>Error: Between MS = 4116.2. df = 24.000												
Cell No.	Sample	{1}	{2}	{3}	{4}	{5}	{6}	{7}	{8}	{9}	{10}	{11}	{12}
1	Control line hypoxia		0.8910	0.0000	0.0000	0.0000	0.0000	0.0000	0.0000	0.0118	0.0001	0.0070	0.0075
2	Control LPS hypoxia	0.8910		0.0000	0.0000	0.0000	0.0000	0.0000	0.0000	0.0086	0.0001	0.0050	0.0104
3	Control IFN-α hypoxia	0.0000	0.0000		0.4158	0.0000	0.0000	0.0000	0.0000	0.0000	0.0000	0.0000	0.0000
4	Control LPS+IFN-α hypoxia	0.0000	0.0000	0.4158		0.0000	0.0000	0.0000	0.0000	0.0000	0.0000	0.0000	0.0000
5	EEP pl 25 μg/mL hypoxia	0.0000	0.0000	0.0000	0.0000		0.0092	0.2793	0.0358	0.0000	0.0338	0.0000	0.0004
6	EEP pl 50 μg/mL hypoxia	0.0000	0.0000	0.0000	0.0000	0.0092		0.0006	0.5493	0.0000	0.0000	0.0000	0.0000
7	EEP pl 25 μg/mL+ LPS hypoxia	0.0000	0.0000	0.0000	0.0000	0.2793	0.0006		0.0028	0.0000	0.2638	0.0000	0.0068
8	EEP pl 50 μg/mL+ LPS hypoxia	0.0000	0.0000	0.0000	0.0000	0.0358	0.5493	0.0028		0.0000	0.0002	0.0000	0.0000
9	EEP pl 25 μg/mL+ IFN-α hypoxia	0.0118	0.0086	0.0000	0.0000	0.0000	0.0000	0.0000	0.0000		0.0000	0.8227	0.0000
10	EEP pl 50 μg/mL+ IFN-α hypoxia	0.0001	0.0001	0.0000	0.0000	0.0338	0.0000	0.2638	0.0002	0.0000		0.0000	0.0812
11	EEP pl 25 μg/mL+ LPS+IFN-α hypoxia	0.0070	0.0050	0.0000	0.0000	0.0000	0.0000	0.0000	0.0000	0.8227	0.0000		0.0000
12	EEP pl 50 μg/mL+ LPS+IFN-α hypoxia	0.0075	0.0104	0.0000	0.0000	0.0004	0.0000	0.0068	0.0000	0.0000	0.0812	0.0000	

Table S7. Statistical Comparison of IL-9 Levels between Experimental Groups Treated with Ethanolic Extract of Propolis (EEP) in Glioma Cells under Hypoxic Conditions (LSD Post Hoc Test)

	LSD test; variable IL-9<br>Probabilities for Post Hoc Tests<br>Error: Between MS = 7.6759. df = 24.000												
Cell No.	Sample	{1}	{2}	{3}	{4}	{5}	{6}	{7}	{8}	{9}	{10}	{11}	{12}
1	Control line hypoxia		0.5715	0.3520	0.0835	0.2637	0.4812	0.9625	0.8128	0.9624	0.5435	0.3284	0.4539
2	Control LPS hypoxia	0.5715		0.1409	0.0256	0.5734	0.8885	0.5403	0.7411	0.5402	0.2457	0.1292	0.1944
3	Control IFN-α hypoxia	0.3520	0.1409		0.4001	0.0470	0.1090	0.3763	0.2462	0.3763	0.7422	0.9617	0.8527
4	Control LPS+IFN-α hypoxia	0.0835	0.0256	0.4001		0.0070	0.0187	0.0915	0.0519	0.0915	0.2459	0.4270	0.3067
5	EEP pl 25 μg/mL hypoxia	0.2637	0.5734	0.0470	0.0070		0.6716	0.2448	0.3744	0.2448	0.0910	0.0425	0.0687
6	EEP pl 50 μg/mL hypoxia	0.4812	0.8885	0.1090	0.0187	0.6716		0.4529	0.6384	0.4528	0.1955	0.0996	0.1527
7	EEP pl 25 μg/mL+ LPS hypoxia	0.9625	0.5403	0.3763	0.0915	0.2448	0.4529		0.7765	0.9999	0.5748	0.3515	0.4822
8	EEP pl 50 μg/mL+ LPS hypoxia	0.8128	0.7411	0.2462	0.0519	0.3744	0.6384	0.7765		0.7765	0.4006	0.2280	0.3269
9	EEP pl 25 μg/mL+ IFN-α hypoxia	0.9624	0.5402	0.3763	0.0915	0.2448	0.4528	0.9999	0.7765		0.5749	0.3516	0.4823
10	EEP pl 50 μg/mL+ IFN-α hypoxia	0.5435	0.2457	0.7422	0.2459	0.0910	0.1955	0.5748	0.4006	0.5749		0.7063	0.8859
11	EEP pl 25 μg/mL+ LPS+IFN-α hypoxia	0.3284	0.1292	0.9617	0.4270	0.0425	0.0996	0.3515	0.2280	0.3516	0.7063		0.8152
12	EEP pl 50 μg/mL+ LPS+IFN-α hypoxia	0.4539	0.1944	0.8527	0.3067	0.0687	0.1527	0.4822	0.3269	0.4823	0.8859	0.8152	

Table S8. Multivariate Analysis of the Effect of Ethanolic Extract of Propolis (EEP) on Cytokine Expression in Glioma Cells under Hypoxic Conditions

	Multivariate Tests of Significance<br>Sigma-restricted parameterization<br>Effective hypothesis decomposition					
Effect	Test	Value	F	Effect	Error	p
Intercept	Wilks	0.002983	1058.565	6	19.0000	0.00
Sample	Wilks	0.000080	7.837	66	107.1221	0.00

Table S9. Statistical Comparison of IL-6 Levels between Experimental Groups Treated with Quercetin in Glioma Cells under Hypoxic Conditions (LSD Post Hoc Test)

	LSD test; variable IL-6<br>Probabilities for Post Hoc Tests<br>Error: Between MS = 1217E2. df = 24.000												
Cell No.	Sample	{1}	{2}	{3}	{4}	{5}	{6}	{7}	{8}	{9}	{10}	{11}	{12}
1	Control line hypoxia (Q)		0.1928	0.0809	0.0000	0.3067	0.4785	0.5049	0.4639	0.3144	0.5627	0.9129	0.4579
2	Control LPS hypoxia (Q)	0.1928		0.6339	0.0001	0.0254	0.0504	0.0550	0.0479	0.7574	0.0659	0.2308	0.5636
3	Control IFN-α hypoxia (Q)	0.0809	0.6339		0.0004	0.0085	0.0179	0.0197	0.0169	0.4345	0.0240	0.0998	0.2962
4	Control LPS+IFN-α hypoxia (Q)	0.0000	0.0001	0.0004		0.0000	0.0000	0.0000	0.0000	0.0001	0.0000	0.0000	0.0000
5	quercetin 25 μg/mL hypoxia	0.3067	0.0254	0.0085	0.0000		0.7483	0.7164	0.7666	0.0492	0.6514	0.2594	0.0846
6	quercetin 25 μg/mL + LPS hypoxia	0.4785	0.0504	0.0179	0.0000	0.7483		0.9661	0.9808	0.0933	0.8953	0.4144	0.1534
7	quercetin 25 μg/mL + IFN-α hypoxia	0.5049	0.0550	0.0197	0.0000	0.7164	0.9661		0.9469	0.1012	0.9290	0.4387	0.1652
8	quercetin 25 μg/mL + LPS + IFN-α hypoxia	0.4639	0.0479	0.0169	0.0000	0.7666	0.9808	0.9469		0.0891	0.8763	0.4011	0.1470
9	quercetin 50 μg/mL hypoxia	0.3144	0.7574	0.4345	0.0001	0.0492	0.0933	0.1012	0.0891		0.1195	0.3683	0.7871
10	quercetin 50 μg/mL + LPS hypoxia	0.5627	0.0659	0.0240	0.0000	0.6514	0.8953	0.9290	0.8763	0.1195		0.4922	0.1924
11	quercetin 50 μg/mL + IFN-α hypoxia	0.9129	0.2308	0.0998	0.0000	0.2594	0.4144	0.4387	0.4011	0.3683	0.4922		0.5258
12	quercetin 50 μg/mL + LPS + IFN-α hypoxia	0.4579	0.5636	0.2962	0.0000	0.0846	0.1534	0.1652	0.1470	0.7871	0.1924	0.5258	

Table S10. Statistical Comparison of VEGF Levels between Experimental Groups Treated with Quercetin in Glioma Cells under Hypoxic Conditions (LSD Post Hoc Test)

	LSD test; variable VEGF<br>Probabilities for Post Hoc Tests<br>Error: Between MS = 1525.1. df = 24.000												
Cell No.	Sample	{1}	{2}	{3}	{4}	{5}	{6}	{7}	{8}	{9}	{10}	{11}	{12}
1	Control line hypoxia (Q)		0.3345	0.4681	0.0765	0.0238	0.0755	0.1215	0.0645	0.1175	0.2946	0.7721	0.0035
2	Control LPS hypoxia (Q)	0.3345		0.8066	0.0091	0.1662	0.3914	0.5408	0.3501	0.0154	0.0508	0.4956	0.0003
3	Control IFN-α hypoxia (Q)	0.4681	0.8066		0.0161	0.1068	0.2736	0.3940	0.2417	0.0267	0.0830	0.6607	0.0006
4	Control LPS+IFN-α hypoxia (Q)	0.0765	0.0091	0.0161		0.0003	0.0011	0.0021	0.0009	0.8223	0.4435	0.0424	0.1768
5	quercetin 25 μg/mL hypoxia	0.0238	0.1662	0.1068	0.0003		0.5839	0.4273	0.6390	0.0005	0.0019	0.0445	0.0000
6	quercetin 25 μg/mL + LPS hypoxia	0.0755	0.3914	0.2736	0.0011	0.5839		0.8029	0.9369	0.0019	0.0073	0.1307	0.0000
7	quercetin 25 μg/mL + IFN-α hypoxia	0.1215	0.5408	0.3940	0.0021	0.4273	0.8029		0.7424	0.0036	0.0132	0.2018	0.0001
8	quercetin 25 μg/mL + LPS + IFN-α hypoxia	0.0645	0.3501	0.2417	0.0009	0.6390	0.9369	0.7424		0.0016	0.0061	0.1130	0.0000
9	quercetin 50 μg/mL hypoxia	0.1175	0.0154	0.0267	0.8223	0.0005	0.0019	0.0036	0.0016		0.5859	0.0673	0.1186
10	quercetin 50 μg/mL + LPS hypoxia	0.2946	0.0508	0.0830	0.4435	0.0019	0.0073	0.0132	0.0061	0.5859		0.1851	0.0401
11	quercetin 50 μg/mL + IFN-α hypoxia	0.7721	0.4956	0.6607	0.0424	0.0445	0.1307	0.2018	0.1130	0.0673	0.1851		0.0017
12	quercetin 50 μg/mL + LPS + IFN-α hypoxia	0.0035	0.0003	0.0006	0.1768	0.0000	0.0000	0.0001	0.0000	0.1186	0.0401	0.0017	

Table S11. Statistical Comparison of PDGF-BB Levels between Experimental Groups Treated with Quercetin in Glioma Cells under Hypoxic Conditions (LSD Post Hoc Test)

	LSD test; variable PDGF-BB<br>Probabilities for Post Hoc Tests<br>Error: Between MS = 23.403. df = 24.000												
Cell No.	Sample	{1}	{2}	{3}	{4}	{5}	{6}	{7}	{8}	{9}	{10}	{11}	{12}
1	Control line hypoxia (Q)		0.4608	0.8381	0.1687	0.0138	0.0457	0.0038	0.0790	0.2225	0.4108	0.9685	0.4000
2	Control LPS hypoxia (Q)	0.4608		0.3486	0.5094	0.0686	0.1871	0.0216	0.2887	0.0567	0.1257	0.4376	0.1213
3	Control IFN-α hypoxia (Q)	0.8381	0.3486		0.1170	0.0086	0.0295	0.0023	0.0524	0.3061	0.5344	0.8690	0.5217
4	Control LPS+IFN-α hypoxia (Q)	0.1687	0.5094	0.1170		0.2281	0.4979	0.0864	0.6816	0.0133	0.0334	0.1575	0.0320
5	quercetin 25 μg/mL hypoxia	0.0138	0.0686	0.0086	0.2281		0.5884	0.5866	0.4194	0.0007	0.0019	0.0126	0.0018
6	quercetin 25 μg/mL + LPS hypoxia	0.0457	0.1871	0.0295	0.4979	0.5884		0.2823	0.7872	0.0026	0.0071	0.0421	0.0068
7	quercetin 25 μg/mL + IFN-α hypoxia	0.0038	0.0216	0.0023	0.0864	0.5866	0.2823		0.1825	0.0002	0.0005	0.0034	0.0004
8	quercetin 25 μg/mL + LPS + IFN-α hypoxia	0.0790	0.2887	0.0524	0.6816	0.4194	0.7872	0.1825		0.0050	0.0133	0.0731	0.0128
9	quercetin 50 μg/mL hypoxia	0.2225	0.0567	0.3061	0.0133	0.0007	0.0026	0.0002	0.0050		0.6816	0.2372	0.6960
10	quercetin 50 μg/mL + LPS hypoxia	0.4108	0.1257	0.5344	0.0334	0.0019	0.0071	0.0005	0.0133	0.6816		0.4332	0.9843
11	quercetin 50 μg/mL + IFN-α hypoxia	0.9685	0.4376	0.8690	0.1575	0.0126	0.0421	0.0034	0.0731	0.2372	0.4332		0.4220
12	quercetin 50 μg/mL + LPS + IFN-α hypoxia	0.4000	0.1213	0.5217	0.0320	0.0018	0.0068	0.0004	0.0128	0.6960	0.9843	0.4220	

Table S12. Statistical Comparison of IL-10 Levels between Experimental Groups Treated with Quercetin in Glioma Cells under Hypoxic Conditions (LSD Post Hoc Test)

	LSD test; variable IP-10<br>Probabilities for Post Hoc Tests<br>Error: Between MS = 2199.7. df = 24.000												
Cell No.	Sample	{1}	{2}	{3}	{4}	{5}	{6}	{7}	{8}	{9}	{10}	{11}	{12}
1	Control line hypoxia (Q)		0.8271	0.0000	0.0000	0.8559	0.7691	0.9255	0.7493	0.0000	0.0000	0.0000	0.0000
2	Control LPS hypoxia (Q)	0.8271		0.0000	0.0000	0.6895	0.6094	0.9006	0.5914	0.0000	0.0001	0.0000	0.0000
3	Control IFN-α hypoxia (Q)	0.0000	0.0000		0.0000	0.0000	0.0000	0.0000	0.0000	0.4002	0.0000	0.5935	0.0025
4	Control LPS+IFN-α hypoxia (Q)	0.0000	0.0000	0.0000		0.0000	0.0000	0.0000	0.0000	0.0000	0.0000	0.0000	0.0000
5	quercetin 25 μg/mL hypoxia	0.8559	0.6895	0.0000	0.0000		0.9107	0.7833	0.8901	0.0000	0.0000	0.0000	0.0000
6	quercetin 25 μg/mL + LPS hypoxia	0.7691	0.6094	0.0000	0.0000	0.9107		0.6989	0.9792	0.0000	0.0000	0.0000	0.0000
7	quercetin 25 μg/mL + IFN-α hypoxia	0.9255	0.9006	0.0000	0.0000	0.7833	0.6989		0.6798	0.0000	0.0000	0.0000	0.0000
8	quercetin 25 μg/mL + LPS + IFN-α hypoxia	0.7493	0.5914	0.0000	0.0000	0.8901	0.9792	0.6798		0.0000	0.0000	0.0000	0.0000
9	quercetin 50 μg/mL hypoxia	0.0000	0.0000	0.4002	0.0000	0.0000	0.0000	0.0000	0.0000		0.0001	0.1750	0.0186
10	quercetin 50 μg/mL + LPS hypoxia	0.0000	0.0001	0.0000	0.0000	0.0000	0.0000	0.0000	0.0000	0.0001		0.0000	0.0352
11	quercetin 50 μg/mL + IFN-α hypoxia	0.0000	0.0000	0.5935	0.0000	0.0000	0.0000	0.0000	0.0000	0.1750	0.0000		0.0006
12	quercetin 50 μg/mL + LPS + IFN-α hypoxia	0.0000	0.0000	0.0025	0.0000	0.0000	0.0000	0.0000	0.0000	0.0186	0.0352	0.0006	

Table S13. Statistical Comparison of MCP-1 Levels between Experimental Groups Treated with Quercetin in Glioma Cells under Hypoxic Conditions (LSD Post Hoc Test)

	LSD test; variable MCP-1<br>Probabilities for Post Hoc Tests<br>Error: Between MS = 15539.. df = 24.000												
Cell No.	Sample	{1}	{2}	{3}	{4}	{5}	{6}	{7}	{8}	{9}	{10}	{11}	{12}
1	Control line hypoxia (Q)		0.0385	0.3080	0.0001	0.5288	0.9092	0.0209	0.6860	0.9106	0.2445	0.7876	0.5893
2	Control LPS hypoxia (Q)	0.0385		0.2623	0.0229	0.1341	0.0489	0.7792	0.0877	0.0302	0.3289	0.0672	0.0115
3	Control IFN-α hypoxia (Q)	0.3080	0.2623		0.0015	0.6909	0.3635	0.1652	0.5331	0.2594	0.8808	0.4493	0.1252
4	Control LPS+IFN-α hypoxia (Q)	0.0001	0.0229	0.0015		0.0006	0.0001	0.0421	0.0003	0.0001	0.0022	0.0002	0.0000
5	quercetin 25 μg/mL hypoxia	0.5288	0.1341	0.6909	0.0006		0.6052	0.0791	0.8201	0.4590	0.5847	0.7171	0.2471
6	quercetin 25 μg/mL + LPS hypoxia	0.9092	0.0489	0.3635	0.0001	0.6052		0.0269	0.7713	0.8210	0.2918	0.8765	0.5140
7	quercetin 25 μg/mL + IFN-α hypoxia	0.0209	0.7792	0.1652	0.0421	0.0791	0.0269		0.0500	0.0162	0.2128	0.0376	0.0059
8	quercetin 25 μg/mL + LPS + IFN-α hypoxia	0.6860	0.0877	0.5331	0.0003	0.8201	0.7713	0.0500		0.6060	0.4408	0.8923	0.3484
9	quercetin 50 μg/mL hypoxia	0.9106	0.0302	0.2594	0.0001	0.4590	0.8210	0.0162	0.6060		0.2037	0.7030	0.6684
10	quercetin 50 μg/mL + LPS hypoxia	0.2445	0.3289	0.8808	0.0022	0.5847	0.2918	0.2128	0.4408	0.2037		0.3664	0.0946
11	quercetin 50 μg/mL + IFN-α hypoxia	0.7876	0.0672	0.4493	0.0002	0.7171	0.8765	0.0376	0.8923	0.7030	0.3664		0.4205
12	quercetin 50 μg/mL + LPS + IFN-α hypoxia	0.5893	0.0115	0.1252	0.0000	0.2471	0.5140	0.0059	0.3484	0.6684	0.0946	0.4205	

Table S14. Statistical Comparison of IL-9 Levels between Experimental Groups Treated with Quercetin in Glioma Cells under Hypoxic Conditions (LSD Post Hoc Test)

	LSD test; variable IL-9<br>Probabilities for Post Hoc Tests<br>Error: Between MS = 14.025. df = 24.000												
Cell No.	Sample	{1}	{2}	{3}	{4}	{5}	{6}	{7}	{8}	{9}	{10}	{11}	{12}
1	Control line hypoxia (Q)		0.0099	0.0297	0.0001	0.0294	0.0127	0.0022	0.0259	0.0079	0.3526	0.0012	0.0017
2	Control LPS hypoxia (Q)	0.0099		0.6290	0.0671	0.6319	0.9143	0.5338	0.6732	0.9240	0.0761	0.3918	0.4709
3	Control IFN-α hypoxia (Q)	0.0297	0.6290		0.0241	0.9967	0.7068	0.2735	0.9507	0.5635	0.1852	0.1860	0.2336
4	Control LPS+IFN-α hypoxia (Q)	0.0001	0.0671	0.0241		0.0243	0.0539	0.2104	0.0276	0.0810	0.0009	0.3059	0.2474
5	quercetin 25 μg/mL hypoxia	0.0294	0.6319	0.9967	0.0243		0.7099	0.2753	0.9540	0.5663	0.1839	0.1873	0.2351
6	quercetin 25 μg/mL + LPS hypoxia	0.0127	0.9143	0.7068	0.0539	0.7099		0.4665	0.7531	0.8392	0.0938	0.3365	0.4085
7	quercetin 25 μg/mL + IFN-α hypoxia	0.0022	0.5338	0.2735	0.2104	0.2753	0.4665		0.3005	0.5977	0.0203	0.8118	0.9202
8	quercetin 25 μg/mL + LPS + IFN-α hypoxia	0.0259	0.6732	0.9507	0.0276	0.9540	0.7531	0.3005		0.6055	0.1666	0.2063	0.2577
9	quercetin 50 μg/mL hypoxia	0.0079	0.9240	0.5635	0.0810	0.5663	0.8392	0.5977	0.6055		0.0630	0.4456	0.5307
10	quercetin 50 μg/mL + LPS hypoxia	0.3526	0.0761	0.1852	0.0009	0.1839	0.0938	0.0203	0.1666	0.0630		0.0118	0.0162
11	quercetin 50 μg/mL + IFN-α hypoxia	0.0012	0.3918	0.1860	0.3059	0.1873	0.3365	0.8118	0.2063	0.4456	0.0118		0.8903
12	quercetin 50 μg/mL + LPS + IFN-α hypoxia	0.0017	0.4709	0.2336	0.2474	0.2351	0.4085	0.9202	0.2577	0.5307	0.0162	0.8903	

Table S15. Multivariate Analysis of the Quercetin on Cytokine Expression in Glioma Cells under Hypoxic Conditions

	Multivariate Tests of Significance<br>Sigma-restricted parameterization<br>Effective hypothesis decomposition					
Effect	Test	Value	F	Effect	Error	p
Intercept	Wilks	0.002367	1334.668	6	19.0000	0.00
Sample	Wilks	0.000137	6.929	66	107.1221	0.00
